# Cognitive perspectives on maintaining physicians’ medical expertise: III. Strengths and weaknesses of self-assessment

**DOI:** 10.1186/s41235-023-00511-z

**Published:** 2023-08-30

**Authors:** Scott H. Fraundorf, Zachary A. Caddick, Timothy J. Nokes-Malach, Benjamin M. Rottman

**Affiliations:** 1 Learning Research and Development Center, University of Pittsburgh, 3420 Forbes Ave., Pittsburgh, PA 15260 USA; 2 Department of Psychology, University of Pittsburgh, 3420 Forbes Ave., Pittsburgh, PA 15260 USA

**Keywords:** Medical expertise, Metacognition, Self-assessment

## Abstract

Is self-assessment enough to keep physicians’ cognitive skills—such as diagnosis, treatment, basic biological knowledge, and communicative skills—current? We review the cognitive strengths and weaknesses of self-assessment in the context of maintaining medical expertise. Cognitive science supports the importance of accurately self-assessing one’s own skills and abilities, and we review several ways such accuracy can be quantified. However, our review also indicates a broad challenge in self-assessment is that individuals do not have direct access to the strength or quality of their knowledge and instead must infer this from heuristic strategies. These heuristics are reasonably accurate in many circumstances, but they also suffer from systematic biases. For example, information that feels easy to process in the moment can lead individuals to overconfidence in their ability to remember it in the future. Another notable phenomenon is the Dunning–Kruger effect: the poorest performers in a domain are also the least accurate in self-assessment. Further, explicit instruction is not always sufficient to remove these biases. We discuss what these findings imply about when physicians’ self-assessment can be useful and when it may be valuable to supplement with outside sources.

## Significance statement

Providing high-quality care requires practicing physicians to assess their own knowledge and skills: when judging whether a tentative diagnosis is appropriate, when deciding whether they need to refer a patient to a specialist, or when selecting what skills and materials to study and practice. The present review captures both the strengths and weaknesses of self-assessment, especially as it could be applied to the context of maintaining and updating medical expertise. We show that self-assessment can be reasonably accurate, and we discuss how this could be leveraged in maintaining physicians’ medical expertise. However, we also highlight some systematic biases and errors in self-assessment, which point to a need for additional, external sources of feedback and guidance.

## Introduction

Physicians’ ability to accurately self-assess their knowledge is likely to be critical to multiple aspects of acquiring and retaining expert performance over time, such as deciding what material to study (and how long to study that material) for continuing certification program assessments, deciding among CME options, and deciding whether to look up additional information for making a decision about an individual patient. Self-assessing knowledge is also critical for deciding whether to refer a patient to a sub-specialist versus treating a patient oneself.

Here, we review what cognitive science suggests about the nature of self-assessment: what it is, why it is important, and how it can be measured. We consider both ways in which self-assessment is accurate as well as its systematic biases and weakness, and we describe theoretical perspectives that account for both. We discuss what may be needed to improve self-assessment before highlighting open questions and proposing relevant future studies.

This article is part of a collection of five articles in this special issue focused on how physicians maintain medical expertise across their careers. We take the approach of a narrative review, not systematic, because it covers a wide variety of topics. To situate the strength of the evidence and claims made, we attach evidence levels (EL) to in-text citations for empirical claims (See Table 1). Evidence levels range from 1 to 6, with 1 being the strongest evidence (meta-analyses) and 6 being the weakest (opinion papers).

**Table 1:** Evidence levels for in-text citations for empirical claims

Evidence level	Type of work
1	Quantitative meta-analysis
2	Narrative review
3	Multiple original experiments/randomized controlled trials (RCTs)
4	Single original experiment/RCT
5	Correlational or quasi-experimental study
6	Opinion paper

## What is self-assessment?

The notion of *self-assessment* has been criticized in the literature on medical expertise for being poorly defined (Eva & Regehr, 2005). It is true that self-assessment is a multifaceted construct and can refer to related but distinct processes. We thus begin by introducing the framework of Nelson and Narens (1990, EL: 2), which has been extremely influential within cognitive psychology. This framework identifies two processes relevant to self-assessment. First, people must *monitor*, or assess their current knowledge and level of performance. For example, when deciding whether they have sufficient expertise to treat a patient versus refer them elsewhere, a physician might monitor their expertise by judging whether they can bring relevant information to mind, remembering their experiences treating similar patients, and/or mentally enumerating their areas of medical expertise. Second, people must *control* their activities, or choose learning and performance strategies informed by this knowledge of their strengths and weaknesses. For example, based on this assessment of expertise, the physician might treat the patient with their current knowledge, look up additional information, or refer the patient to a specialist. Together, these processes are termed *metacognition*, or reasoning about one’s own thinking and knowledge.

Research from cognitive psychology supports the claim that accurate self-assessment matters for learning: There is evidence both that (a) monitoring is causally related to decisions about learning and that (b) those decisions in turn alter the type and amount of learning that occurs. For instance, monitoring of knowledge appears to have a causal role in determining what learners study and how much time they spend on it (Metcalfe & Finn, 2008, EL: 3; Metcalfe, 2009, EL: 2; Thiede et al., 2003, EL: 4). Across domains and participant groups, learners often choose to study material they have judged that they do not know as well (the *discrepancy reduction* strategy; Dunlosky & Hertzog, 1997, EL: 5; Son & Metcalfe, 2000, EL: 2; c.f., Metcalfe & Kornell, 2003, EL: 3; Miller, 2005, EL: 3). In turn, decisions about what to study matters for long-term retention: Learners who focus their study time on difficult material end up with more overall knowledge than learners who spend on their time on easy material (Tullis & Benjamin, 2011, EL: 5; c.f., Nelson & Leonesio, 1988, EL: 5). More broadly, good awareness of one’s own thinking (i.e., metacognition) predicts academic success even when controlling for general intelligence (Ohtani & Hisasaka, 2018, EL: 1).

A key implication for the retention of medical expertise is that physicians’ ability to self-assess has direct consequences for their behavior. If physicians do not accurately monitor their knowledge, they will make poor decisions about what to study for continuing certification program assessments and what to review in everyday practice. Indeed, physician overconfidence has been linked to diagnostic errors (Berner & Graber, 2008, EL: 2).

## Monitoring accuracy has two components

Before we can draw any conclusions about how accurately people can self-assess their knowledge, we first must consider how accuracy can be measured. Laboratory studies have assessed the monitoring component of metacognition by having participants: (a) complete some task (e.g., answering science questions) and (b) rate their level of performance. A critical question in research on monitoring has been how closely perceived performance aligns with actual performance: If self-assessments are accurate, then higher confidence should predict a higher probability of correct responding, and lower confidence a lower probability.

Methodologists (e.g.,Juslin et al., 1996; Lichtenstein & Fischhoff, 1977; Murphy, 1973; Nelson, 1996; Nelson & Dunlosky, 1991; Schraw, 2009; Yates, 1982) have delineated how monitoring accuracy can be assessed in terms of both calibration and resolution. *Calibration* (or *absolute accuracy*) is how well a learner can predict their overall level of performance. For example, if I predict that I will get a B average in my classes this term, do I earn a B average (good calibration), or do I earn an A or C average (poorer calibration)? Calibration identifies whether learners are overconfident, underconfident, or appropriately confident in their skills. Good calibration would be demonstrated if, for instance, a physician who estimated that their initial diagnoses were incorrect 10% of the time was indeed incorrect 10% of the time (rather than more or less). This kind of monitoring would be important when physicians judge whether their knowledge is “good enough”; that is, is their current knowledge good enough to provide effective care for the patient population that they see, or do they need to look up additional information or acquire additional training?

Assessing calibration requires learners to provide judgments on a scale that can be directly compared to objective criterion performance. For example, to measure calibration on tests where objective accuracy is measured on a 0–100% scale, the confidence scale would also need to refer to the probability of correct responding (e.g., on a 0–100% Likert scale, or smaller intervals such as “0%”, “25%”, “50%”, “75%”, or “100%”). This would represent a change for many assessments of medical expertise, where confidence is often assessed using more subjective terms, such as “somewhat confident” or “very confident.” Unfortunately, such ratings do not permit a true assessment of whether a learner is overconfident or underconfident because there is no objective definition of what it means to be “somewhat confident.” However, there would be several potential advantages to collecting confidence judgments in a format that can assess calibration—most critically, the ability to give physicians feedback on whether they are overconfident or underconfident, as well as asking novel research questions, such as how calibration varies across performance outcomes.

A second type of monitoring accuracy is *resolution* (or *relative accuracy*), which is how well a learner can identify their relative strengths and weaknesses, such as their areas of expertise, or the particular patients for whom their judgments are more or less likely to be corrected. For example, if I think I am more knowledgeable about diabetes than thyroid problems, is that true (good resolution), or am I in fact better with the thyroid than diabetes (bad resolution)? In self-assessing medical expertise, good resolution would be demonstrated physicians expressed more confidence in the specific situations were indeed better at. This kind of monitoring is important when physicians decide which patients need further consideration and when they choose which topics to study for continuing certification program assessments or which CME activities to participate in.

Researchers have debated which form of monitoring is most important for physicians. Some (Omron et al., 2018; Zwaan & Hautz, 2019) have argued that a particular problem for physicians is poor calibration—specifically, overconfidence. Physicians may be overconfident in their skills because even when they make an error (e.g., misdiagnose a patient or provide incorrect treatment), they often does not get adequate feedback about this because the patient may recover anyway, go to another treatment center, or die (see Rottman et al., 2023, for further discussion). Indeed, meta-analysis and review suggest overconfidence is widespread and physicians’ self-monitoring is poorly calibrated (Berner & Graber, 2008, EL: 2; Gordon, 1991: EL 1). On the other hand, Eva, Regehr, and colleagues have argued (Regehr et al., 1996; Eva & Regehr, 2005, 2007, 2011) that, in practice, physicians rarely need to assess their overall level of performance or functioning; rather, it is more important to identify the specific cases for which physicians need to slow down and devote more care, a capacity that seems to align with resolution. Our view is that it is likely both calibration (“do I know enough about hypertension?”) and resolution (“do I know more about hypertension or diabetes?”) would be valuable for physicians, but it is clear more work in this space is needed, especially to directly compare these two capabilities. Indeed, one reason for the lack of clarity on this point may be that not all work has recognized that there are separate measures of metacognitive monitoring that quantify different things.

## Metacognitive monitoring can be reasonably accurate

### Confidence predicts accuracy

Can learners monitor their learning per both standards discussed above? In many cases, monitoring can be reasonably accurate, though imperfect: On average, higher confidence in one’s cognitive skills predicts a somewhat greater probability that one is correctly answering a question or correctly completing a task, both in terms of calibration and discrimination. This is true across multiple types of performance. For example, people can monitor their *episodic memory*—knowledge of specific events, such as an individual patient’s symptoms and diagnosis—with reasonable accuracy such that, generally speaking, the more confident someone is in their memory, the more likely it is to be accurate (e.g., Banks, 2000, EL: 5; Benjamin et al., 2009, EL: 5; Egan, 1958, EL: 5; Tweed et al., 2020, EL: 5; Wickelgren & Norman, 1966, EL: 5; Wixted, 2007, EL: 5; Wixted & Wells, 2017, EL: 5). It is also broadly true for semantic knowledge—that is, more general world knowledge, such as the name of a nation’s capital or the appropriate drugs to treat a particular syndrome (Berdie, 1971: EL 5; Goldsmith & Koriat, 2007, EL: 5; Koriat & Goldsmith, 1996, EL: 5; Metcalfe, 1986, EL: 5; Smith & Clark, 1993, EL: 5), as well as particular *categories* of knowledge (e.g., science vs. history, or ankle problems vs. knee problems; Eva & Regehr, 2007, EL: 4). Indeed, even when learners are unable to bring desired information to mind in the moment, they can accurately monitor whether they are likely to be able to retrieve that information in the future (the *feeling of knowing*; Freedman & Landauer, 1966, EL: 5; Gruneberg & Monks, 1974, EL: 5; Hart, 1965, EL: 5; Hart, 1967, EL: 5; Metcalfe, 1986, EL: 5; Nelson & Narens, 1980a, EL: 5; Nelson & Narens, 1980b, EL: 5; Smith & Clark, 1993, EL: 5).

**Fig. 1:**
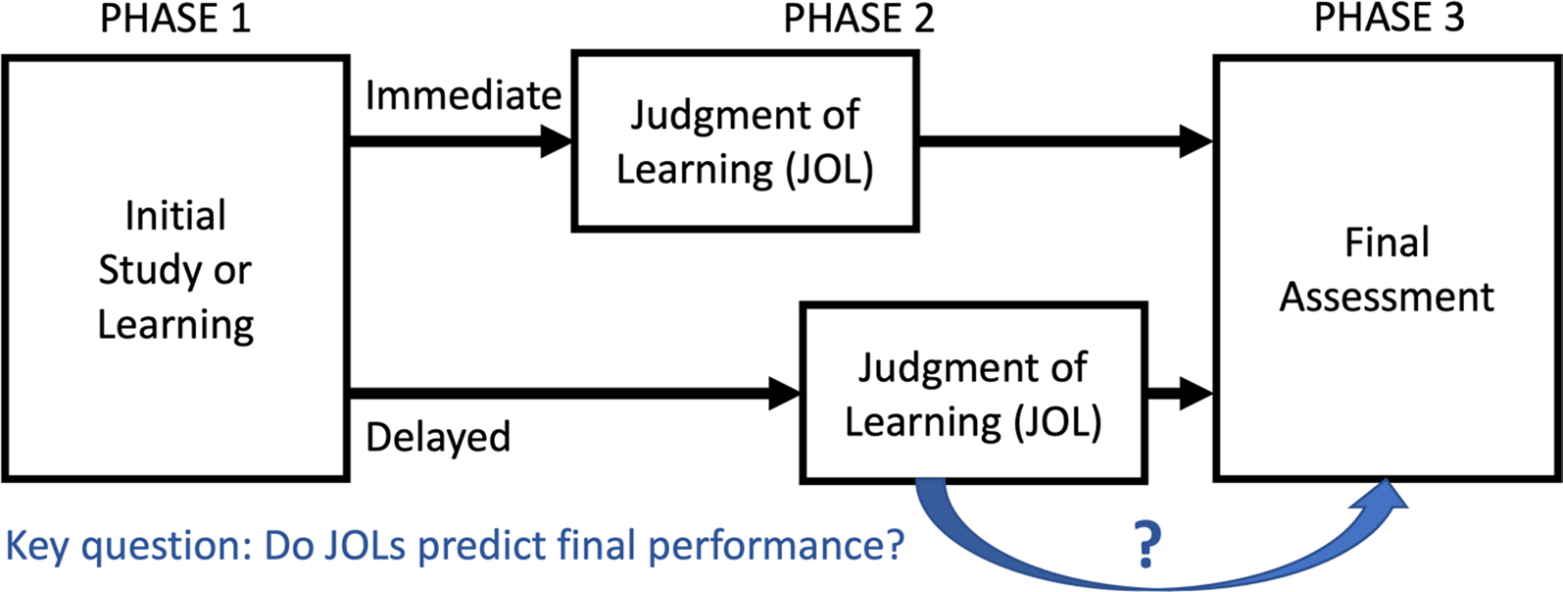
Schematic design of the typical judgment-of-learning (JOL) study procedure with immediate JOLs (top row) and delayed JOLs (bottom row)

Of course, when physicians choose what to study or practice, they need to evaluate not just their immediate knowledge, but their ability to retain, access, and use that information in the future. Laboratory studies have tested this ability, too, by adapting the confidence-monitoring paradigm reviewed above into the *judgments of learning* paradigm (Fig. 1). In this paradigm, learners first study novel material and/or review existing knowledge for a future test or task. These materials similarly vary across studies and include science facts, examples of to-be-learned categories (e.g., different species of birds), and word pairs, among others. After studying each item, the learner provides—either immediately or after a delay—a judgment of learning (JOL), which is an assessment of how likely they are to be able to respond correctly *on the future test*. For example, learners would rate how likely they are to remember a science fact, or to be able to classify the species depicted in a photograph of a bird. Lastly, the learner takes some form of test or assessment on the material. When JOLs are made at a delay after initial learning, they can strongly predict later performance (Nelson & Dunlosky, 1991, EL: 5); meta-analysis indicates a 0.75 correlation between delayed JOLs and later performance (Rhodes & Tauber, 2011, EL: 1). However, when JOLs are made immediately after learning, their predictive power is somewhat reduced (a correlation of 0.42; Rhodes & Tauber, 2011, EL: 1), for reasons we discuss later.

The implication of these laboratory studies is that physicians are likely to be able to self-assess their skills and knowledge with a moderate, though imperfect, degree of accuracy. This conclusion has been echoed by several reviews of the medical literature (Gordon, 1991: EL 1; Davis et al., 2006: EL 2), which have found that physicians’ self-assessments do predict their objective performance, but only weakly to moderately. (Note, however, that these measures did not always distinguish calibration from discrimination.) Indeed, the ability to accurately judge whether one knows something can be challenging in the health sciences: Learners’ accuracy in self-assessing their knowledge about healthcare varies widely, but on average is fairly poor (Gordon, 1991, EL: 2), especially for clinical performance as compared to factual knowledge. Where calibration diverges from the ideal, it is often in the direction of physicians being overconfident in their diagnoses, decision-making, and assessments (Berner & Graber, 2008, EL: 2; Gordon, 1991: EL 1).

Thus, depending on one’s perspective, the glass of self-assessment is either half empty or half full. On the one hand, the imperfections of metacognitive monitoring—including some systematic biases that we review below—mean that self-assessment alone is likely insufficient. On the other, given that learners do have some ability to monitor themselves, that capability could be leveraged in designing longitudinal continuing certification program assessments; for instance, by allowing physicians some control over which topics to be tested on. Physicians may be able to choose and practice the particular topics that they struggle with (assuming that the early assessments are fairly low-stakes). Additionally, physicians may have some insights into what topics are not relevant for their practice. For example, if an orthopedist has restricted their practice to adult hips and knees, it may not make sense to ask questions about pediatric problems or about adult ankles, feet, elbows, shoulders, or spines.

Would such learner control of which materials to study be helpful? Laboratory studies find that learner control of which materials to study is superior to allocating study time equally or based on normative difficulty (Koriat et al., 2006, EL: 3; Mazzoni & Cornoldi, 1993, Experiment 3, EL: 4; Tullis & Benjamin, 2011, EL: 3). However, a meta-analysis of classroom studies (Karich et al., 2014, EL: 1) found weak to nonexistent evidence that such practices benefit students. Given the ambiguity of the available evidence, it is an open question whether physicians’ own self-assessments are more or less accurate at identifying topics that should be studied compared to an algorithm based on their prior performance.

### People can control reporting in multiple ways

Above, we have shown that people can—to some degree—self-assess the accuracy of a specific task response. Another important kind of monitoring is to determine whether and how one should respond at all. For example, physicians must decide whether to diagnose a patient based on their current knowledge or instead consult a colleague or external resource. Indeed, Ward et al. (2002) argue that it is more important for physicians to know when to stop and seek external resources (such as peers or the medical literature) than it is to have precise accuracy in monitoring their cognitive skills. Here, we evaluate in turn each of several response strategies: declining to respond, adjusting the grain size of a response, looking up information, and seeking help from others.

Koriat and Goldsmith (1994, EL: 3) developed a two-phase laboratory procedure to test whether people can accurately self-assess whether to respond at all. In an initial phase, participants answer general world-knowledge questions (e.g., *What is the chemical process responsible for the formation of glucose in the plant cell?*) but have the option to withhold responses; payment for participation is structured such that participants lose money for incorrect responses but not for withholding responses. In the second phase, participants revisit each question and are required to respond. This permits comparison between participants’ accuracy when allowed to withhold responses versus when required to respond. Critically, questions for which participants withhold responses in phase 1 are much less likely to be answered correctly in phase 2, indicating that people were successfully able to self-assess what they did not know (Goldsmith & Koriat, 1999, EL: 3; Kelley & Sahakyan, 2003, EL: 5; Koriat & Goldsmith, 1994, EL: 5; Koriat & Goldsmith, 1996, EL: 5; Koriat et al., 2008, EL: 5; Goldsmith & Koriat, 2007, EL: 5). Similarly, Eva and Regehr (2011, EL: 3) found that when learners were provided with an opportunity to skip a test question that was outside their knowledge set, they chose to skip items that they would have answered incorrectly.

A less drastic adjustment than withholding a response entirely is to provide an estimate or judgment at a different *grain size*. For example, imagine a physician trying to estimate how long an infection would take to clear up. The physician could provide a specific estimate (5 weeks), a narrow range (4 to 6 weeks), or a wider range (2 to 8 weeks). People can also self-assess the appropriate grain size to some degree. The two-phase procedure described above yields similar evidence for effective metacognition when, rather than being given the option to withhold responses, participants are instead allowed to control the grain size of reporting, e.g., reporting that the Berlin Wall fell in the interval 1985 *to* 1995 when less confident versus reporting 1989 when more confident (Goldsmith et al., 2005, EL: 5; Goldsmith et al., 2002, EL: 5; Koriat et al., 2008, EL: 5; Neisser, 1988; Yaniv & Foster, 1997, EL: 5).

Two other ways that people can adjust their responses are to withhold a response until they can consult an external resource (e.g., the internet; Ferguson et al., 2015, EL: 3) or another person for help. Here, people’s behavior may align less closely with their metacognitive monitoring; although people are broadly more likely to consult external aids when less confident (Cotler et al., 1970, EL: 5; Nelson & Fyfe, 2019; EL: 5; Undorf et al., 2021, EL: 3), they sometimes do not seek help even when low in confidence (Undorf et al., 2021, EL: 3). One reason for this may be that seeking external help incurs additional costs, such as requiring more time or—in the case of asking another person—social judgment from one’s peers or supervisors (Halabi & Nadler, 2017, EL: 2; Karabenick & Gonida, 2018, EL: 3; Nadler, 1991, EL: 3; Nadler, 2017, EL: 3; Nadler & Chernyak-Hai, 2014, EL: 3, but see Miranda Lery Santos et al., 2020, EL: 4, for null effects of the time taken to request help). Such negative consequences of help-seeking may be particularly strong for individuals from socially disadvantaged groups, for whom help-seeking may be viewed as reinforcing negative stereotypes of inability or dependence (Halabi et al., 2016, EL: 5; Halabi & Nadler, 2017, EL: 2; Nadler, 2017, EL: 3; Nadler & Chernyak-Hai, 2014, EL: 3). However, these conclusions stem from studies with varied forms of help or external resources, and there is a need to study help-seeking behavior with the specific kinds of resources most apt to be used by physicians (e.g., UpToDate).

Nevertheless, the broad need to self-assess when to report versus when to “look it up” leads to the speculative suggestion that it may be beneficial for assessments of medical expertise to additionally assess whether physicians can judiciously employ such responses and perhaps even to train this metacognitive skill. In the proposed studies below, we describe one method that might be used for such an assessment.

## Metacognitive monitoring is subject to systematic biases

Although monitoring can be reasonably accurate in some cases, as we discuss above, research has also documented several important errors and biases in self-assessment. We review several key biases before turning to theoretical accounts that can explain them.

### Learners underestimate both learning and forgetting

People underestimate the degree to which their cognitive skills will change in the future. On the one hand, people greatly underestimate how much they will forget between the time they learn information and the time that they need to use it (Koriat et al., 2004, EL: 3), likely because recently acquired knowledge feels strong and salient in the moment. On the other hand, when learners start with low initial knowledge, they *under*estimate how much they can learn in the future because that knowledge initially feels difficult and inaccessible. Even as people practice and gain skill, their JOLs tend to reflect their initial struggles (the *underconfidence-with-practice effect*; Koriat, 2008b, EL: 3; Koriat et al., 2002; c.f., Serra & Dunlosky, 2005, EL: 3). Even when people do expect their skills to improve, they rely too greatly on their initial experiences in forming expectations: People who are initially the most adept at a task tend to forecast their skills will improve the most (the *performance heuristic*; Critcher & Rosenzweig, 2014, EL: 3), even though in fact such people have the least room to improve.

The tendency for people to treat their present state of skill or knowledge as if it will continue forever has been termed the *stability bias* (Kornell & Bjork, 2009, EL: 3). This bias is likely to influence physicians’ self-assessment of medical expertise in two ways: First, physicians may underestimate how much they may forget after their initial training, and so the accuracy of their self-assessment years later may be inflated in the absence of external feedback. Second, they may conversely underestimate the degree to which their skills and knowledge are amenable to learning and practice—even in their current areas of weakness and even when practices need to update to conform to advances in medicine. This may lead physicians to forego beneficial training or review unless externally prompted to do so.

A corollary to the fact that people underestimate forgetting is the observation that self-assessment is better at a delay. One of the most robust phenomena in monitoring is the *delayed-JOL* effect (Rhodes & Tauber, 2011, EL: 1; Nelson & Dunlosky, 1991, EL: 5): JOLs made immediately after initial learning show low resolution, but *delayed JOLs* made sometime after later initial learning (e.g., during a second, later study session) predict memory quite accurately. This difference can be explained in terms of the ease-of-processing heuristic we discuss below (Begg et al., 1989, EL: 5). Immediately after studying, knowledge is still active in the learner’s immediate working (or short-term) memory^1^ and feels fluent and accessible. But, over time, the contents of working memory are lost, thus rendering immediate fluency a poor index of later performance (Benjamin et al., 1998, EL: 3). By comparison, what comes to mind sometime after training is much more diagnostic of long-term retention (Begg et al., 1989, EL: 5). An implication for long-term retention is that self-assessments are best performed separately from learning or feedback; confidence ratings asked immediately after a CME course, or immediately after feedback on a continuing certification program question, are unlikely to be indicative of a physician’s long-term expertise.

### Learners sometimes evaluate information sources based on superficial fluency

Learners sometimes judge the reliability or utility of information sources based on relatively superficial sources of fluency (Alter & Oppenheimer, 2009, EL: 2; Oppenheimer, 2008, EL: 2). For example, students judge themselves as learning more from a lecture when the teacher stands upright and makes eye contact, even when this does not influence actual learning (Carpenter et al., 2013, EL: 3; see also Fiechter et al., 2018, EL: 3).

This bias suggests that fluency of use is important to consider in designing any continuing certification platform. There may be some cases in which *dis*fluency is desirable insofar as it can engender more analytic, “System 2” thinking (e.g., Alter, 2013, EL: 2; Alter et al., 2007, EL: 3; Alter et al., 2013, EL: 6; Diemand-Yauman et al., 2011, EL: 3; Keysar et al., 2012, EL: 3), although this claim has also been disputed (Meyer et al., 2015, EL: 1; Thompson et al., 2013, EL: 3; Yue et al., 2013, EL: 3). However, that may be less relevant to a longitudinal assessment, which is intended for assessment and learning, rather than optimizing in-the-moment decision-making. Thus, all other things being equal, fluency is likely to help create physician buy-in for continuing certification: Physicians will likely perceive that they are learning more if the system presents a fluent, easy-to-use experience.

### Learners neglect optimal learning conditions

Learners often fail to appreciate optimal learning conditions (Finn & Tauber, 2015, EL: 2). For example, categorization tasks (e.g., learning to categorize a set of symptoms as one disease versus another) are often learned better by intermixing (*interleaving*) the to-be-learned categories rather than presenting them one at a time (*blocking*; Bjork & Bjork, 2019, EL: 3; Brunmair & Richter, 2019, EL: 1; c.f., Kurtz & Hovland, 1956, EL: 4). However, given the choice, learners often block practice and view this as superior to interleaving (Kirk-Johnson et al., 2019, EL: 3; Kornell & Bjork, 2008a, EL: 3; Kornell et al., 2010, EL: 3; Wahlheim et al., 2012, EL: 3; Yan et al., 2016, EL: 3; Zulkiply et al., 2012, EL: 3). This apparent metacognitive error has been attributed to the fact that blocked practice creates a sense of fluency in the moment even though it is less effective for long-term learning and retention (Kirk-Johnson et al., 2019, EL: 3; Yan et al., 2016, EL: 3).

Similarly, although retrieval practice potentiates long-term retention (as we review elsewhere), learners typically judge tested materials as *less* well-learned than restudied materials (Kirk-Johnson et al., 2019, EL: 5; Roediger & Karpicke, 2006, EL: 5) and choose restudying over retrieval practice (Kirk-Johnson et al., 2019, EL: 5). And, generating or creating to-be-learned material (e.g., through a fill-in-the-blank prompt) is more effective than simply passively reading it (the *generation effect*; Slamecka & Graf, 1978, EL: 3). However, because of the additional effort associated with generation, learners perceive generated material as *less* well-learned (Besken & Mulligan, 2014, EL: 3).

A general principle is thus that learners often mistake the initial effort required by effective study strategies (Schmidt & Bjork, 1992, EL: 3) as a sign those strategies are ineffective and consequently do not choose to use them (Kirk-Johnson et al., 2019, EL: 5). This implies that physicians left to study on their own may be studying in less effective or less efficient ways than they might if they are explicitly directed.

### Accessing external knowledge may be misperceived as having knowledge

Modern information technology allows physicians—and others—to quickly access external sources of information (e.g., via UpToDate.com). But, several studies have found that accessing information from the internet or other external sources (e.g., books) can create the illusion of internally possessing that knowledge (Eliseev & Marsh, 2023, EL: 3; Fisher et al., 2015, EL: 3; Hamilton & Yao, 2018, EL: 3; Pieschl, 2021, EL: 4; Siler et al., 2022, EL: 3; Ward, 2021, EL: 3), though this finding has not always been replicated (Ferguson et al., 2015, EL: 4). Thus, if physicians have access to external resources when self-assessing, they may overestimate the extent of their own personal knowledge.

This misattribution may be relatively benign if the resources that physicians access during self-assessment are the same that they will use on the job; in this case, self-assessment would still accurately reflect later performance. Indeed, as we have discussed above, knowing when to consult external resources is an important metacognitive skill, and—-in an era of easily accessible information technology—it may be important to know how and where to locate external information than to memorize it oneself (Marsh & Rajaram, 2019, EL: 2; Sparrow et al., 2011, EL: 3). But, it does imply that the only external resources provided during the self-assessment should be those that physicians will later use (e.g., UpToDate, WebMD, guidelines); otherwise, self-assessments are likely to be inaccurately influenced by those external resources.

### Learners stop studying too soon

Learners often terminate study too quickly: They study too few items (Murayama et al., 2016, EL: 3), and, among the items they *do* study, they do not devote sufficient time or repetitions to optimize learning (Karpicke, 2009, EL: 3; Kornell & Bjork, 2008b, EL: 3). Some of this behavior may simply reflect the fact that learners will not persist indefinitely at studying in the face of other, competing activities (Kurzban et al., 2013, EL: 6). However, it may also reflect errors in self-monitoring insofar as learners do not always recognize when learning can be increased by continuing to study (Murayama et al., 2016, Experiment 5, EL: 4). This metacognitive error has been argued to relate to the stability bias: Once learners have learned material sufficiently well enough to respond correctly in the moment, they terminate study because they do not recognize that their cognitive skills will decline over time (Kornell & Bjork, 2008b, EL: 3). Thus, external assessment may potentially be useful for inducing additional, beneficial practice beyond what learners would naturally engage in.

### Poor performers overestimate their performance

**Fig. 2:**
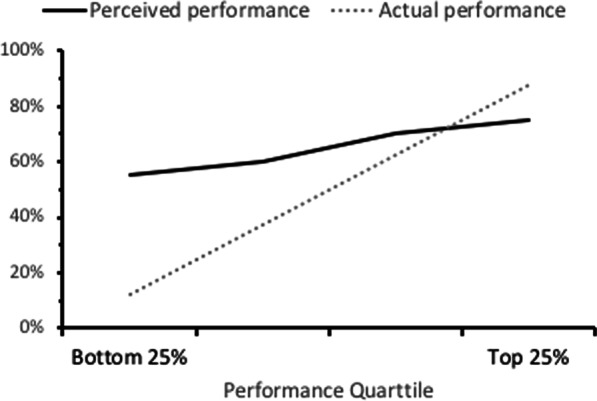
Prototypical Dunning–Kruger effect (not representing data from any specific study)

Another important bias that has been identified in the calibration of metacognitive monitoring is the *Dunning–Krueger effect* (Fig. 2): People with low skill often greatly overestimate their performance (Dunning et al., 2003, EL: 5; Kruger & Dunning, 1999, EL: 4). That is, those who perform poorly in a domain are often unaware they are doing poorly; they are “unskilled and unaware.” (By contrast, high performers if anything *underrate* their performance; Kruger & Dunning, 1999, EL: 5). This phenomenon has been found across many domains including college social science (Dunning et al., 2003; EL: 5), formal logic (Kruger & Dunning, 1999, EL: 4), humor (Kruger & Dunning, 1999, EL: 5), English grammar (Kruger & Dunning, 1999, EL: 5), face recognition (Zhou & Jenkins, 2020; EL: 5), and—most critically for our purposes—medicine (Berner & Graber, 2008, EL: 2; Davis et al., 2006, EL: 2; Hodges et al., 2001: EL 5; Parker et al., 2004, EL: 5; Sears et al., 2014, EL: 2).

What causes the Dunning–Kruger effect? In most domains, the knowledge required for effective metacognitive monitoring is often the same as, or at least similar to, the knowledge for effective cognitive performance (Kruger & Dunning, 1999, EL: 5; Dunning, 2011, EL: 2). For instance, imagine students factoring quadratic equations in an algebra class. To check if they have the right answer, students need to know the same rules they would use to solve the problem; a student who has learned the wrong rules will both produce the wrong answer *and* be unable to tell that answer is wrong. Low skill thus results in a “double curse” of both inaccurate performance and inaccurate self-assessment. An implication for medical expertise is that physicians low in domain knowledge may be unaware of this fact and unable to correctly self-assess their lack of expertise.

### Other factors can influence what learners choose to study

Choices in self-regulated study are guided by variables beyond those that would maximize learning and retention. Learners also preferentially practice material that they find *interesting*, regardless of how well they have learned it, and even when they know that learning is necessary for an upcoming task (Son & Metcalfe, 2000, EL: 3). Learners also fall into habits and routines of studying, such as reviewing material in the order it was originally presented, regardless of what needs the most practice (Ariel et al., 2009, EL: 3; Ariel et al., 2011, EL: 3; Macaluso et al., 2022, EL: 4).

Thus, while there are advantages to customization, leaving the areas of physicians’ continuing study wholly up to physicians (e.g., for CME courses or for a continuing certification program) may be insufficient because physicians in some cases may defer to what they find interesting or what they routinely do rather than where they may need the most continuing education.

## Theoretical mechanisms

Why are self-assessments not always objectively correct, and what accounts for the biases discussed above? Cognitive psychology has generally rejected a *direct-access* view of metamnemonic monitoring (Koriat, 1995, EL: 5; Koriat, 1997, EL: 5): Learners do not have the ability to directly “read off” the strength of their memory traces. Some of the starkest evidence against direct access comes from circumstances—such as very difficult questions for which the most common response is incorrect—that reverse the confidence-accuracy relationship, so that answers given more confidently are actually *less* likely to be correct (Koriat, 2008a, EL: 5). This would not be possible if self-assessment were an objective assessment of knowledge.

Instead, cognitive psychology suggests an *inferential* view of metamemory (Schwartz et al., 1997, EL: 2; Koriat, 1997, EL: 5): Learners make an “informed guess” about their skill and knowledge based on various heuristics that are often, but not always, correct (Benjamin et al., 1998, EL: 6). For example, a strong predictor of memory confidence is simply the amount of information that comes to mind, whether it is right or wrong (Koriat, 1993, EL: 5). This could be explained by a heuristic whereby people base their confidence judgments on the amount of information that comes to mind. This strategy will generally produce accurate self-assessments because people do often bring to mind more information about material they know well, but it is not guaranteed to be correct.

The inferential nature of metamnemonic monitoring implies that not *all* self-assessment will be accurate and that physicians may benefit from external feedback on their accuracy. Further, while heuristic strategies are often accurate—which is likely why they exist in the first place—there are edge cases where they fail to produce optimal outcomes, which could explain some of the biases discussed above.

In particular, one heuristic that may explain many of the biases reviewed above is what Kornell et al., (2011, EL: 3) have termed the *ease-of-processing heuristic*: Material that is experienced as subjectively fluent or easy to process in the moment is judged as better understood and learned (Alter & Oppenheimer, 2009, EL: 2; Begg et al., 1989, EL: 3; Oppenheimer, 2008, EL: 2; see also the closely related heuristic of *easily learned, early remembered*: Koriat, 2008b, EL: 4). Researchers have argued for the prevalence of this heuristic in learners’ judgments on the basis a wealth of experiments in which manipulations of fluency that are irrelevant to actual learning are nevertheless shown to affect JOLs. For instance, learners give higher JOLs to items that are written in a larger font (Kornell et al., 2011, EL: 3; Rhodes & Castel, 2008; EL: 3), that are louder (Rhodes & Castel, 2009, EL: 3), that have greater visual clarity (Besken, 2016, EL: 3; Besken & Mulligan, 2013, EL: 3), even though each of these variables was unrelated to genuine memory within the respective experiments. Conversely, learners may disregard features that *do* matter for retention but that do not enhance immediate fluency (Sungkhasettee et al., 2011, EL: 3), such as the planned number of future study opportunities (Kornell et al., 2011, EL: 3). Not all of these effects necessarily reflect implicit effects of fluency; in some cases, they might reflect learners’ explicit beliefs that, for instance, text printed in large type is indeed more memorable (Besken et al., 2019, EL: 3; Mueller et al., 2014, EL: 3; Undorf & Zimdahl, 2019, EL: 3), so it remains an important ongoing debate the extent to which biases stem from an ease-of-processing heuristic versus learners’ genuine beliefs (correct or incorrect) about what variables influence learning. Nevertheless, processing fluency has been observed to influence JOLs even in cases where verbalizable beliefs do not have such an influence (Undorf et al., 2017, EL: 3; Yang et al., 2018, EL: 3); indeed, in at least some cases, fluency has been shown to directly mediate effects on JOLs (Undorf et al., 2017, EL: 3; Yang et al., 2018, EL: 3). Therefore, the ease-of-processing heuristic appears to account for at least some, though not all, biases in metacognitive monitoring.

We emphasize that the ease-of-processing heuristic is likely to be accurate in many cases: Often, material that feels fluent and effortless *is* better learned (Benjamin et al., 1998, EL: 3; Koriat, 2008b, EL: 4). Nevertheless, it can also explain many of the biases reported above. Because learners use their current cognitive accessibility as a proxy for long-term learning, they underestimate both how much that accessibility may decline with forgetting or increase with study, yielding the stability bias. And, because initial fluency is an imperfect index of what contributes to long-term learning (Benjamin et al., 1998, EL: 3; Soderstrom & Bjork, 2015, EL: 3), a reliance on initial fluency may lead learners to misperceive optimal learning conditions. The ease-of-processing heuristic can also explain why information from external sources, like the internet, can be mistaken for personal knowledge: The ability to rapidly access knowledge online can create a feeling of cognitive ease that learners may mistake for genuine understanding. Indeed, experimental evidence of the relationship between quick access and a feeling of knowing comes from laboratory studies that manipulated the speed at which web pages loaded in an online search task; the faster the page loaded, the better participants felt they could retain the information (Stone & Storm, 2019, EL: 3).

The ease-of-processing heuristic is likely to have implications in clinical settings. As we have reviewed elsewhere (Caddick et al., 2023), physicians are often quite successful in their clinical decision-making. But because the right answer (e.g., a clinical diagnosis) so often arrives quickly to the mind of the physician (Barrows et al., 1982, EL: 5; Elstein et al., 2013, EL: 5; Gruppen et al., 1988, EL: 5; Pelaccia et al., 2011, EL: 5), they might not always appropriately judge a wrong answer that also arrives quickly and easily.

## Explicit instruction does not remove self-assessment biases

We have reviewed how people often use their subjective, in-the-moment experience as a heuristic to self-assess their knowledge and learning. Such judgments have been termed *non-analytic* because they are not necessarily based on conscious, verbalized introspection (Kelley & Jacoby, 1996, EL: 3).

Perhaps one solution to the biases of these non-analytic judgments would be to simply warn physicians that the accuracy of their self-assessment may be flawed. Indeed, cognitive psychology does suggest that, beyond these non-analytic “gut feelings,” people also hold explicit, verbalizable beliefs about which circumstances favor learning and performance, which can be used as the basis of *analytic* judgments (Fraundorf & Benjamin, 2014, EL: 4; Kelley & Jacoby, 1996, EL: 3; Koriat et al., 2004, EL: 3). For example, some learners may adopt spaced repetition because they have been taught that it is an effective study strategy, regardless of their own experience using this method (Lu & Fraundorf, 2020, EL: 3).

However, self-assessment using explicit, analytic beliefs is not a panacea. First, we cannot assume that people already know the best learning strategies. Non-scientists’ beliefs about effective learning and memory are often inaccurate, as revealed by surveys of the general public (Simons & Chabris, 2011, EL: 5; Simons & Chabris, 2012, EL: 5; Yan et al., 2014a, 2014b, EL: 5), of college students (Hartwig & Dunlosky, 2012, EL: 5; Karpicke et al., 2009, EL: 5; McCabe, 2011, EL: 5; Morehead et al., 2016, EL: 5), and even of college instructors (Morehead et al., 2016, EL: 5). For example, most people describe self-testing only as a way to assess their current knowledge and not as a way to potentiate learning (Hartwig & Dunlosky, 2012, EL: 5; Kornell & Bjork, 2007, EL: 5; McCabe, 2011, EL: 5; Morehead et al., 2016, EL: 5; Yan et al., 2014a, 2014b, EL: 5); thus, they are unlikely to spontaneously make use of the testing effect. Why do people have such mistaken beliefs about effective learning? One reason may be that they were simply never taught otherwise: About two-thirds of the U.S. population report they never received formal instruction on how best to learn (Yan et al., 2014a, 2014b, EL: 5).

Second, even when learners *do* hold accurate analytic beliefs (e.g., they believe that testing potentiates long-term retrieval), those beliefs are not always activated and *used* in self-assessment. For instance, although presumably all adults understand to some degree that information is forgotten over time, people asked to predict how much they will remember a full year later give estimates no different than people asked to predict what they will remember a mere week later. Only when the question specifically uses the word “forgetting” does this belief become activated and influence predictions (Koriat et al., 2004, EL: 3). Similarly, even when people are explicitly told that in-the-moment fluency can be a misleading basis for self-assessment and instructed to disregard it, they are not entirely successful in doing so (e.g., Besken & Mulligan, 2014, EL: 3; Yan et al., 2016, EL: 3).

A key implication for the maintenance of cognitive skills is that we cannot expect physicians to naturally know how best to self-assess or keep their knowledge current. Further, simply instructing physicians on how best to self-assess may be insufficient because even if physicians acquire accurate analytic beliefs (e.g., that testing benefits long-term retention), those beliefs will not always be used in self-assessment. Instead, external prompts for practice and self-assessment may be critical.

## Proposed studies and future directions

### Response scale for confidence judgments

Currently, physicians’ confidence judgments are collected on different scales across various longitudinal assessments. It would be useful to explore the optimal means of assessing confidence. As we discussed above, confidence scales that include some reference to an objective standard of performance (e.g., “75% confident I’m right”) would allow measures of calibration (e.g., overconfidence vs. underconfidence) to be collected and provided as feedback. It would also be useful to determine how many different intervals or categories of confidence can be differentiated by learners—can physicians meaningfully distinguish between, for instance, being “very confident” versus “extremely confident”? This issue is important because, given imprecision in how people translate internal confidence into external ratings (Benjamin et al., 2009: EL 2), a scale with too many categories may in fact decrease the accuracy of confidence ratings (Benjamin et al., 2013: EL 3). Lastly, it may be valuable to determine whether the highest level of confidence (e.g., “I’m virtually certain”) represents a qualitatively distinct state of special accuracy, as proposed by certain dual-process theories of recognition (Parks & Yonelinas, 2007: EL 2; Yonelinas, 1994: EL 3; Yonelinas, 2002: EL 2; c.f., Wixted, 2007: EL 2).

### Autonomy and learning outcomes

Given that people can self-assess their knowledge and skills with reasonable accuracy in many situations, it may be of interest to allow physicians some control over the topics they study. We suggest it would be valuable to investigate how greater autonomy in choosing to-be-learned material affects physicians’ learning outcomes. Individuals could be randomized to groups with varying degrees of control over the learned content (e.g., 25% control of content vs. 75% control), before both groups’ knowledge is tested at a later date. Learning gains could be compared across methods for the chosen material, unchosen material, and overall.

Perhaps it would also make sense to allow physicians to specify which sorts of material they want to study for which reason. For example, they could separately rate which areas are most important for their practice and how confident they are in their knowledge of each area, and the test could then focus on topics that are relevant but for which the physician has lower confidence. Additionally, motivational measures could be assessed to see if increased autonomy leads to increased intrinsic motivation (see Nokes-Malach et al., 2022, for further discussion).

Though there are reasons to hypothesize that autonomy can lead to improved learning—both by increasing motivation and by capitalizing on physicians’ knowledge of their areas of weakness—this is not a certainty. In fact, one study on continuing medical education found that quality of care improved only for CME topics that physicians did *not* prefer to learn about, rather than the ones they did (Sibley et al., 1982, EL: 4). In sum, it is important to study if and how autonomy or self-direction over study topics can improve learning; there are reasons to think that it may help, but also reasons to think that it may not.

### Physician customization and psychometric quality

Longitudinal assessment has two purposes. First, a longitudinal assessment serves as a summative assessment that Diplomates must pass to maintain their certification. It is critical to establish and maintain the quality of the summative aspects that will be used to make pass–fail decisions. The pass–fail decision is often the hurdle that prevents some Diplomates from remaining certified, and in those instances, the test publisher will need firm evidence to justify that decision. In particular, making defensible pass–fail decisions is simplified if there is a high degree of standardization so that all examinees attempting to maintain their certification are responsible for similar content mastery reflecting the certificates they hold.

Second, a longitudinal assessment should also provide formative feedback to help Diplomates continue to improve the breadth, depth, and currency of their medical knowledge throughout their career (an “assessment *for* learning”). At times, this second purpose may be at odds with the first. Consider customization that allow each participant to tailor the assessment (in whole or part) to the areas in which they need or wish to improve. This customization may help provide better formative feedback and give Diplomates a greater sense of relevance to their practice. However, customization can sometimes degrade the fit between the measurement and the intended meaning of the certificate.

Therefore, validity studies and analyses of psychometric quality should continue to be conducted to ensure that quality of the summative component has not been compromised by customization. A few relevant questions include: Is the precision of the participants’ scores sufficient to make defensible pass–fail decisions? Are the number of questions scored for summative purposes sufficient to represent the specialty or subspecialty? If questions are being repeated for spaced repetition, are the scores degraded by the lack of independence?

### Self-assessment versus self-monitoring

Eva and Regehr (2011, EL: 3) propose a distinction between *self-assessment* at the global level (e.g., “How good a physician am I?”) versus *self*-*monitoring* of specific topic areas (e.g., “How much do I know about hypertension?”). In laboratory studies, they found that college students could predict their performance much more accurately for specific questions than at a global level. This distinction is relevant if physicians’ confidence ratings are to be used for any purpose, such as controlling which topics a longitudinal assessment focuses on. At what level of granularity must these confidence ratings be collected to be accurate? We suggest comparing self-assessment accuracy across different levels of granularity. For instance, physicians can be asked to self-assess their competency globally as a physician (the highest level), at a topic level (e.g., hypertension; medium level), and at an item level (e.g., a targeted question about hypertension; the lowest level). The practical question is whether accurate self-assessment can be obtained by querying physicians at a more general level or only at the item level.

### Objective versus comparative self-assessment

Another dimension on which self-assessments vary is whether they are made relative to an *objective* standard (e.g., “What percent correct will you get on this assessment?”) or to a *social* or *comparative* standard (e.g., “How well do you think you will perform on this assessment relative to other doctors?” or “What percentile will you score in?”; Festinger (1954, EL: 2). Some evidence outside medicine suggests that people are more sensitive to their objective standing than their comparative standing (Hoelzl & Rustichini, 2005, EL: 4; Kruger & Burrus, 2004, EL: 3; Moore & Kim, 2003, EL: 3; Windschitl et al., 2003, EL: 3) and, perhaps as a result, are more responsive to objective than comparative feedback (Moore & Klein, 2008, EL: 3). Nevertheless, it would be useful to collect physicians’ self-assessments in both objective and comparative terms to determine which yields more accurate self-assessment.

### Do physicians know when to look it up?

In their practice, physicians have the option of deferring judgment to look up information or refer a patient to a specialist. It may thus be useful to evaluate how accurately physicians can judge when they should consult external resources. This could be tested by adapting the Koriat and Goldsmith (1994) procedure discussed above. In a first encounter with each test item, physicians could be given an option to withhold a response; then, in a second pass through each item, physicians would be required to respond. If physicians can correctly identify when they have insufficient knowledge to answer a question on their own, second-pass accuracy should be lower on the questions where physicians withheld an initial response compared to questions where they volunteered one. Further, given potential differences in when people withhold answers entirely versus request help (Undorf et al., 2021, EL: 3), it would be useful to study when physicians choose to consult an external resource and whether these behaviors indeed improve their accuracy.

### Determine how to create learner buy-in

Learners’ self-assessment of the potential benefits of a longitudinal assessment system is unlikely to be wholly accurate given the biases in self-assessment described above. Further, merely instructing people on desirable learning strategies—such as simply *telling* them that they will learn more from longitudinal assessment—is generally insufficient enough to change beliefs or behavior (McDaniel & Einstein, 2020: EL 2; Yan et al., 2016: EL 3). To guide learners to truly recognize the value of longitudinal assessment and create the most buy-in, more rigorous intervention may be needed to promote accurate self-assessment (Gordon, 1992: EL 2), such as presenting differentiated feedback on performance under different learning conditions (Benjamin, 2003, EL: 3; Tullis et al., 2013, EL: 3; Yan et al., 2016, EL: 3).

## Summary and conclusion

Metacognitive control of learning consists of two processes: (a) monitoring of one’s own knowledge and abilities and (b) control of learning and performance strategies. Prior research supports that accurately self-assessing (monitoring) one’s own abilities and knowledge is important to guiding (controlling) one’s learning and maintaining one’s expertise. For instance, self-assessment is associated with the quality of learning strategies an individual employs and consequently their learning outcomes.

Learners do not appear to have direct access to the strength of their skills or knowledge and instead have only an “informed guess.” These “informed guesses,” although partly accurate, are subject to systematic biases. For example, information or skills that feel easier to process in the moment can lead individuals to overconfidence in how much they will remember in the future. Thus, self-assessments of knowledge immediately after learning tend to be less accurate than delayed judgments. Relatedly, learners often stop studying too soon and underestimate the requisite amount of practice needed to adequately learn and retain target information. The tendency to judge learning based on in-the-moment fluency can also lead to choosing suboptimal learning strategies because those strategies feel more fluent at the time of study.

Another notable bias in the self-assessment literature is the Dunning–Kruger effect, the robust finding—including among physicians—that the poorest performers are the least accurate in their self-assessments and tend to overestimate their actual ability. Conversely, the top performers tend to underestimate their ability, though this bias is not as severe.

Although some preliminary evidence suggests that experiencing different learning conditions with feedback might improve self-assessment accuracy, merely instructing learners about the existence of these biases is not enough to remediate them. Instead, externally guided learning for physicians—including in a longitudinal assessment program—is likely to be critical to retaining and updating cognitive skills.

## Data Availability

Not applicable.

## Footnotes

1 Working memory is a temporary memory system with limited capacity for information and is distinct from long-term memory, where stored information decays relatively little over time.
